# Supplementary material for: Historical Evolutionary Dynamics and Phylogeography Analysis of Transmissible Gastroenteritis Virus and Porcine Deltacoronavirus: Findings from 59 Suspected Swine Viral Samples from China
Source: Int J Mol Sci. 2022 Aug 29;23(17):9786. doi: 10.3390/ijms23179786 (PMC9456201; doi:10.3390/ijms23179786)
Supplement: Supplementary file 1 [file ijms-23-09786-s001.zip › ijms-1857977-supplementary.pdf]

Supplementary material Tables and Figures

Table. S1 Primers for TGEV in this study.

Primers	Sequences	Sites	Length/bp
TGEV-F1	5′- ACTTTTAAAGTAAAGTGAGTGT-3′	1-1957	1957
TGEV-R1	5′- TAACCCTCTCCTGGTGCTAC-3′		
TGEV-F2	5′- GCTGGTATTGAGGCCAAATGCT-3′	1725-3626	1902
TGEV-R2	5′- GGCCTGAAGCTGATAGCAACAA-3′		
TGEV-F3	5′- TGCTGAAGGGACTTCTTCTCAA-3′	3371-6387	3017
TGEV-R3	5′- TGACAACATGCAAAAACCAACT-3′		
TGEV-F4	5′- GCATAAATTAACATGTAACGGTGCT-3′	6062-8971	2910
TGEV-R4	5′- TGTGCCATTTTCCGCAAACC-3′		
TGEV-F5	5′- CTGCTGCTATGGGTACTTTTGT-3′	8698-11635	2938
TGEV-R5	5′- CCATCAAGAGTTGCTGACGC-3′		
TGEV-F6	5′- ACCAGGCTCGTAATGGTGTTC-3′	11308-14273	2966
TGEV-R6	5′- ACTCATTGGAGAGGCGGTAGA-3′		
TGEV-F7	5′- CTGCAACACGCAATGCTACT-3′	14037-16956	2920
TGEV-R7	5′- GCAGGTGGTATGTATTGTTCGC-3′		
TGEV-F8	5′- ATGTTGCTCGGCGTCTTCT-3′	16656-19572	2917
TGEV-R8	5′- GGTAATTTCACTTGTGCACCATA-3′		
TGEV-F9	5′- TACTCTTGGACGATTTTGTGACT-3′	19317-22261	2945
TGEV-R9	5′- CACCAACAGGACTCAACGACA-3′		
TGEV-F10	5′- TGGTCTTGGTATGAAGCGTAGT-3′	21963-24855	2914
TGEV-R10	5′- TCGTCAAGTACAGCATCTACGG-3′		
TGEV-F11	5′- TAGTACAGGTTGCTGTGGATGC-3′	24597-26996	2400
TGEV-R11	5′- TTCCGACCACGGGAATTGG-3′		
TGEV-F12	5′- ATATGTTTGTGAATGACCTCACGTT-3′	25626-28584	2959
TGEV-R12	5′- TTTGTATCACTATCAAAAGGAAA-3′		

Table. S2 Primers for PDCoV in this study.

Primers	Sequences	Sites	Length/bp
PDCoV-F1	5′-ACATGGGGACTAAAGATA-3′	1-2959	2959
PDCoV-R1	5′- GGTTTGTCTTCAACAGCCTGG-3′		

PDCoV-F2	5′- CCGACGAAGACACAGAAGGA-3′	2677-5628	2952
PDCoV-R2	5′- GGTATTTCCTGCCTTGAACGAC-3′		
PDCoV-F3	5′- GCCCCTTCAGACCACTGACT-3′	5309-8389	3081
PDCoV-R3	5′- GATCCTGGTTGGAGCCTTTCA-3′		
PDCoV-F4	5′- TCCAGGTGTAGTTGAGCGATG-3′	8090-11084	2995
PDCoV-R4	5′- AAGGTAGGCTGTCTTAGGGTCT-3′		
PDCoV-F5	5′- CAGTCCTTAACTTGGACCCCC-3′	10852-13867	3016
PDCoV-R5	5′- AGGTTTACCATCATGCTCTGCT-3′		
PDCoV-F6	5′- AACTTCCACCACGACACATCT-3′	13516-16545	3030
PDCoV-R6	5′- TGAGCCCAAGTGACAAAGCAT-3′		
PDCoV-F7	5′- AGCAGCACACGCCATAAAAC-3′	16261-19299	3039
PDCoV-R7	5′- CCTGATGCAACGAGATAGCG-3′		
PDCoV-F8	5′- CTACTCACTTGCGGGTTGGT-3′	19006-22050	3045
PDCoV-R8	5′- ACCGTATCTGGATGACTGAGA-3′		
PDCoV-F9	5′- AGGGCGAGGCATTATCACAC-3′	21791-24290	2500
PDCoV-R9	5′- CCCAAGTAACACGAGTGGTTG-3′		
PDCoV-F10	5′- GAATTCGCTGTTCTGATATACCAGG-3′	23590-25398	1809
PDCoV-R10	5′- TCCATCCCCCCTATAAGCCA-3′		

Table. S3 Identification primers.

Virus	Primer	Sequence	Length/bp
PoRV	PCV3-F	5′-CCCCGGTATTGAATATACCACAGT-3′	333
	PCV3-R	5′-TTTCTGTTGGCCACCCTTTAGT-3′	
PDCoV	PDCoV-F	5′-ATTCTGCTTTGGCTGCTC-3′	354
	PDCoV-R	5′-GGCGGATTTCTGACTGAT-3′	
PEDV	PEDV-F	5′-GCAACAACAGGTCCAGAT-3′	563
	PEDV-R	5′-CTCACGAACAGCCACATT-3′	
PRRSV	PRRSV-F	5′-TGTTGGGGAAGGTCTTGAC-3′	602
	PRRSV-R	5′-CTAGAGACGACCCCATCGTT-3′	
SADS-CoV	SADS -F	5′-CTCAACAGCCGTCACAGTCT-3′	276
	SADS- R	5′-GCTGAACGAGGTCACTGTCA-3′	
TGEV	TGEV-F	5′-TTACAAACTCGCTATCGCATGG-3′	588
	TGEV-R	5′-ACGAGGTCAGTGTCACCAAAATTGG-3′	

Table. S4 TGEV sequences information.

Accession	Country	Host	Collection_Date
AJ271965	USA	pig	2001
DQ201447	China	pig	2004
DQ443743	China	Sus scrofa	2006
DQ811785	USA	pig	2006
DQ811786	USA	porcine	1987
DQ811787	USA	porcine	2006
DQ811788	USA	porcine	2006
DQ811789	USA	porcine	1952
EU074218	China	Sus scrofa	2007
FJ755618	China	pig	1973
HM776941	China	swine	2009
HQ462571	China	Sus scrofa	2010
KC962433	China	Sus scrofa	2012/5/5
KP202848	China	Sus scrofa	2013/3/7
KR270796	USA	pig	2014/7/24
KT696544	China	pig	2012
KU729220	China	Sus scrofa	1998/2/10
KX058075	China	pig	2012
KX083668	China	swine	2015
KX499468	China	porcine	2015/12/22
KX900393	USA	Sus scrofa	2006/12/8
KX900394	USA	Sus scrofa	1988
KX900395	USA	Sus scrofa	2006/11/8
KX900396	USA	Sus scrofa	2006/11/17
KX900397	USA	Sus scrofa	2007/2/8
KX900398	USA	Sus scrofa	2007/2/8
KX900399	USA	Sus scrofa	2007/2/16
KX900400	USA	Sus scrofa	2008/3/6
KX900401	USA	Sus scrofa	2008/4/15
KX900402	Mexico	Sus scrofa	2008/4/17
KX900403	USA	Sus scrofa	2008/4/9
KX900404	USA	Sus scrofa	2012/11/29
KX900405	USA	Sus scrofa	2013/1/4
KX900406	USA	Sus scrofa	2013/2/28
KX900407	USA	Sus scrofa	2013/3/1
KX900408	USA	Sus scrofa	2014/1/17
KX900409	USA	Sus scrofa	2014/1/31
KX900410	USA	Sus scrofa	2014/2/4
KX900411	USA	Sus scrofa	2014/2/5

KY406735	USA	Sus scrofa	2016/11/1
MT522161	China	porcine	2017
MT576083	China	pig	2016/3/1
MZ368889	China	pig	2020/6/24
NC_038861	USA	pig	2000
OM802899	China	swine	2019/7/4
ON859974	China	swine	2021/2/10

Table. S5 PDCoV sequences information.

**Accession**	**Country**	**Host**	**Collection_Date**
JQ065042.2	China	pig	2009
KJ462462	USA	pig	2014/1/31
KJ481931	USA	swine	2014/1/4
KJ567050	USA	pig	2014/2/20
KJ569769	USA	pig	2014/2/13
KJ584355	USA	pig	2014/2/12
KJ584356	USA	pig	2014/2/20
KJ584357	USA	pig	2014/3/7
KJ584358	USA	pig	2014/2/18
KJ584359	USA	pig	2014/2/21
KJ601777	USA	swine	2014/1/8
KJ601778	USA	swine	2014/1/8
KJ601779	USA	swine	2014/1/11
KJ601780	USA	swine	2014/1/26
KJ620016	USA	pig	2014/3/18
KJ769231	USA	swine	2014/3/1
KM012168	USA	pig	2014/3/17
KM820765	South Korea	pig	2014/4/1
KP757890	China	pig	2004/5/24
KP757891	China	pig	2014/12/26
KP757892	China	pig	2014/12/20
KR131621	China	pig	2015/3/1
KR150443	USA	pig	2015/3/24
KR265847	USA	pig	2014/3/6
KR265848	USA	pig	2014/3/14
KR265849	USA	pig	2014/4/2
KR265850	USA	pig	2014/4/2
KR265851	USA	pig	2014/5/13
KR265852	USA	pig	2014/4/21

KR265853	USA	pig	2013/10/14
KR265854	USA	pig	2014/5/21
KR265855	USA	pig	2014/5/21
KR265856	USA	pig	2014/2/23
KR265857	USA	pig	2014/2/23
KR265858	USA	pig	2014/5/6
KR265859	USA	pig	2014/2/11
KR265860	USA	pig	2014/2/5
KR265861	USA	pig	2014/2/5
KR265862	USA	pig	2014/3/26
KR265863	USA	pig	2014/3/27
KR265864	USA	pig	2014/3/14
KR265865	USA	pig	2014/6/5
KT021234	China	porcine	2015/3/20
KT266822	China	pig	2012
KT336560	China	Sus scrofa	2014/11/24
KT381613	USA	pig	2014/5/7
KU051641	Thailand	pig	2015/6/10
KU051649	Thailand	pig	2015/6/30
KU665558	China	Sus scrofa	2014/6/26
KU981059	China	piglet	2015/2/16
KU981060	China	Sus scrofa	2015/4/15
KU981061	China	Sus scrofa	2015/7/18
KU981062	China	Sus scrofa	2015/12/30
KU984334	Thailand	pig	2015/11/1
KX022602	USA	pig	2015/10/15
KX022603	USA	pig	2015/12/18
KX022604	USA	pig	2015/11/27
KX022605	USA	pig	2015/12/21
KX118627	Laos	pig	2016/1/20
KX361343	Thailand	pig	2013/2/1
KX361344	Thailand	pig	2013/3/1
KX361345	Thailand	pig	2015/12/1
KX443143.2	China	Sus scrofa	2016
KX834351	Viet Nam	piglet	2015/10/10
KX834352	Viet Nam	pig	2015/12/8
KX998969	Viet Nam	pig	2015/12/1
KY065120	China	porcine	2016
KY293677	China	piglet	2016/5/23
KY293678	China	piglet	2016/5/23
KY354363	South Korea	porcine	2016/4/1
KY354364	South Korea	porcine	2016/4/1
KY363867	China	swine	2016/3/18
KY363868	China	swine	2016/1/5

KY364365	South Korea	pig	2014/7/1
KY513724	China	pig	2014
KY513725	China	pig	2014
KY926512	South Korea	pig	2016/11/1
LC216914	Hong Kong	Sus scrofa	2014/2/13
LC216915	Hong Kong	Sus scrofa	2014/2/13
LC260038	Japan	Sus scrofa	2014/5/1
LC260039	Japan	Sus scrofa	2014/5/1
LC260040	Japan	Sus scrofa	2014/5/1
LC260041	Japan	Sus scrofa	2014/5/1
LC260042	Japan	Sus scrofa	2014/3/1
LC260043	Japan	Sus scrofa	2014/8/1
LC260044	Japan	Sus scrofa	2014/12/1
LC260045	Japan	Sus scrofa	2016/9/1
MF041982	China	pig	2016/12/23
MF095123	China	Sus scrofa	2017/2/15
MF280390	China	swine	2016
MF431742	China	pig	2015
MF431743	China	pig	2014
MF642322	China	Tibetan pig	2016/8/1
MF642323	China	Tibetan pig	2016/8/1
MF642324	China	Tibetan pig	2017/4/1
MF642325	China	Tibetan pig	2017/3/1
MF948005	China	porcine	2017/8/10
MG242062	China	Sus scrofa	2017
MG832584	China	pig	2016/7/1
MG837130	South Korea	pig	2016/11/1
MG837131	South Korea	pig	2016/11/1
MH025762	China	swine	2016
MH025763	China	swine	2016
MH025764	China	swine	2016
MH708123	China	pig	2018/3/20
MH708124	China	pig	2018/3/20
MH708125	China	pig	2018/3/20
MH715491	China	swine	2016
MK005882	China	porcine	2018/3/1
MK211169	China	porcine	2017/12/24
MK330604	China	porcine	2017/2/1
MK330605	China	porcine	2018/1/1
MK355396	China	Sus scrofa	2016/2/20
MK359104	China	pig	2018
MK572803	China	Sus scrofa	2017/6/1
MK625638	China	swine	2018/10/1
MK625639	China	swine	2018/10/1

MK625640	China	swine	2018/10/1
MK625641	China	swine	2018/10/1
MK993519	China	porcine	2019/1/1
MN025260	China	porcine	2017/1/18
MN173779	China	Sus scrofa	2018
MN173780	China	Sus scrofa	2018
MN173781	China	Sus scrofa	2018
MN173782	China	Sus scrofa	2018
MN249445	China	Sus scrofa	2017/12/11
MN520190	China	Sus scrofa	2018/3/1
MN520191	China	Sus scrofa	2019
MN520192	China	Sus scrofa	2018/8/1
MN520193	China	Sus scrofa	2018
MN520194	China	Sus scrofa	2018/10/1
MN520195	China	Sus scrofa	2018/3/1
MN520196	China	Sus scrofa	2018/3/1
MN520197	China	Sus scrofa	2018/4/1
MN520198	China	Sus scrofa	2019
MN520199	China	Sus scrofa	2018
MN520200	China	Sus scrofa	2018
MN520201	China	Sus scrofa	2018
MN520202	China	Sus scrofa	2018/5/1
MN520203	China	Sus scrofa	2018/11/1
MN520204	China	Sus scrofa	2018/5/1
MN520205	China	Sus scrofa	2019/3/1
MN520206	China	Sus scrofa	2019/3/1
MN520207	China	Sus scrofa	2018/10/1
MN520208	China	Sus scrofa	2018/7/1
MN520209	China	Sus scrofa	2019/1/1
MN942260	China	swine	2015
MT227371	Peru	porcine	2019/10/1
MT260149	China	swine	2018/5/25
MT260150	China	swine	2018/6/5
MT263013	China	swine	2017
MT663769	China	Sus_scrofa	2019/7/23
MW196362	USA	swine	2019
MW685622	Haiti	Homo sapiens	2014/12/15
MW685623	Haiti	Homo sapiens	2015/3/16
MW685624	Haiti	Homo sapiens	2015/4/13
MW854634	Taiwan	pig	2015/6/1
MZ291567	USA	swine	2014
MZ772936	China	pig	2016
MZ802772	Thailand	Sus scrofa	2016/9/1
MZ802773	Thailand	Sus scrofa	2016/9/1

MZ802774	Thailand	Sus scrofa	2016/9/1
MZ802775	Thailand	Sus scrofa	2016/1/1
MZ802776	Viet Nam	Sus scrofa	2016/3/1
MZ802777	Thailand	Sus scrofa	2016/5/1
MZ802955	China	swine	2020/9/1
NC_039208	China	pig	2010
OK546242	China	pig	2020
OM256446	China	pig	2021/11/1
ON859973	China	swine	2021/1/20

Table. S6 Model test for TGEV.

**Molecular clock model**	**Coalescent tree prior**	**Log marginal likelihood**
Strict clock	Constant Size	-56031.54399
Strict clock	Bayesian Skyline	-56040.1100
Uncorrelated relaxed clock	Constant Size	-55753.9165
Uncorrelated relaxed clock	Bayesian Skyline	-55748.9444

Table. S7 Model test for PDCoV.

**Molecular clock model**	**Coalescent tree prior**	**Log marginal likelihood**
Strict clock	Constant Size	-77896.8149
Strict clock	Bayesian Skyline	-77871.5288
Uncorrelated relaxed clock	Constant Size	-77321.6228
Uncorrelated relaxed clock	Bayesian Skyline	-77304.2854

Table. S8 BSSVS analysis data for TGEV

**From**	**To**	**Bayes factor**	**Posterior probability**	**Migration rate**
China	Mexico	1.56306	0.5600488	0.724
China	USA	3.66677	0.7491389	2.354
Mexico	China	1.42569	0.5372736	0.722
Mexico	USA	3.24304	0.7253638	0.782
USA	China	11.16239	0.9008999	0.631

USA	Mexico	11050.891	1.0	0.679

Table. S9 BSSVS analysis data for PDCoV

From	To	Bayes factor	Posterior probability	Migration rate
USA	China	31.98861	0.882247	1.318
USA	Haiti	31.85074	0.881797753	0.94
USA	Japan	28494.5972	1	2.494
USA	South Korea	195.02328	0.978576779	1.753
USA	Peru	1.87648	0.305318	0.917
China	Haiti	31.03558	0.895205993	0.487
China	Japan	0.99054	0.188314607	0.969
China	South Korea	0.98763	0.187865169	0.965
China	Peru	1.03558	0.195205993	0.967
China	USA	95.02328	0.948782013	0.53
Haiti	China	0.92156	0.17752809	0.9
Haiti	Japan	1.11680	0.207340824	0.915
Haiti	South Korea	1.14237	0.211086	0.935
Haiti	Peru	0.97023	0.185168539	0.906
Haiti	USA	1.35269	0.240599251	0.915
Japan	China	1.31195	0.23505618	0.935
Japan	Haiti	1.16818	0.214831461	0.916
Japan	South Korea	10.86533	0.717902622	1.146
Japan	Peru	1.66653	0.280749	0.933
Japan	USA	2.26545	0.346666667	0.938
South Korea	China	1.02868	0.194157303	0.91
South Korea	Haiti	1.04151	0.196104869	0.927
South Korea	Japan	1.01395	0.191910112	0.944
South Korea	Peru	8.09444	0.654681648	1.049
South Korea	USA	1.55730	0.267265918	0.907
Peru	China	0.74703	0.148913858	0.86
Peru	Haiti	0.59720	0.119594	0.918
Peru	Japan	1.40941	0.260749	0.91
Peru	South Korea	1.28384	0.214757	0.934
Peru	USA	0.25629	0.056629213	0.88

Table. S10 Distance and migration rate for TGEV

From	To	Migration rate	Distance(km)
China	USA	2.354	11172
Mexico	USA	0.782	3169
USA	China	0.631	11172
USA	Mexico	0.679	3169

Table. S11 Distance and migration rate for PDCoV

From	To	Migration rate	Distance(km)
China	Haiti	0.487	13906
China	USA	0.53	11172
Japan	South Korea	1.146	1151
South Korea	Peru	1.049	15898
USA	China	1.318	11172
USA	Haiti	0.94	3034
USA	Japan	2.494	10162
USA	South Korea	1.753	10751

Table. S12 Bayesian Evaluation of Temporal Signal (BETS) for TGEV

Molecular clock model	Tip dates	Log marginal likelihood
Strict clock	Sampling dates	-56020.2033
Strict clock	No sampling dates	-56672.2109
Uncorrelated relaxed clock	Sampling dates	-55021.5419
Uncorrelated relaxed clock	No sampling dates	-55029.7912

Table. S13 Bayesian Evaluation of Temporal Signal (BETS) for PDCoV

Molecular clock model	Tip dates	Log marginal likelihood
Strict clock	Sampling dates	-75774.2396
Strict clock	No sampling dates	-1.91E+58

Uncorrelated relaxed clock	Sampling dates	-75083.8023
Uncorrelated relaxed clock	No sampling dates	-75089.7951

Table S14. Positive selection analysis of the TGEV and PDCoV S protein.

Organism	Site	SLAC (P value)	FEL (P value)	MEME (P value)	FUBAR (Post.prob)
TGEV	218	0.0922	0.0079	0	\
PDCoV	110		0.0653	0.09	0.979
	123		0.0362	0.05	0.974
	137		0.0209	0.03	0.984
	527	\	0.0404	0.06	0.969
	630		0.0290	0.02	0.978
	642		0.0609	0.05	0.99
	1016		0.0515	0.07	0.96

Figure S1 Temporal signal testing based on an ML tree using TempEst for TGEV and PDCoV

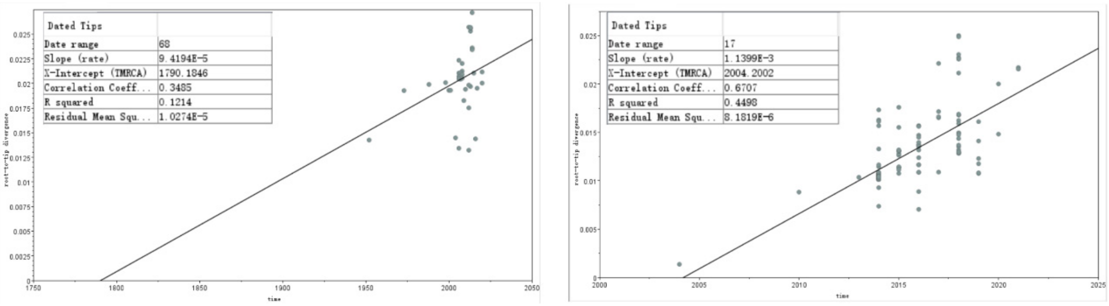

Figure S2 Plot of distance and migration rate for TGEV and PDCoV

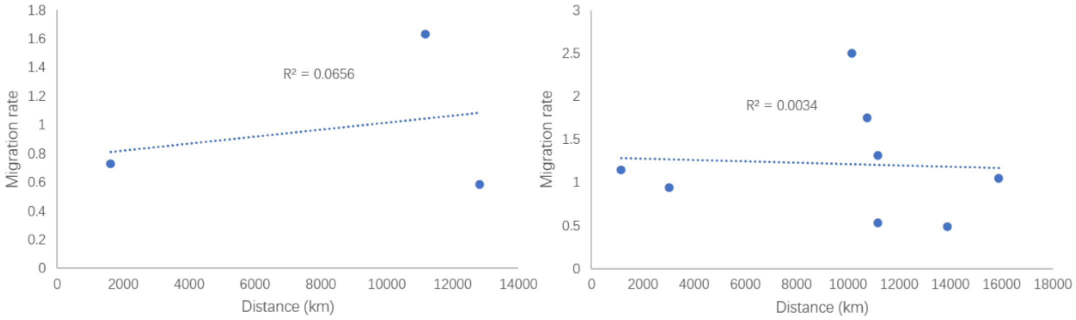

Figure S3 The recombination analysis of TGEV full genomes by RDP4.

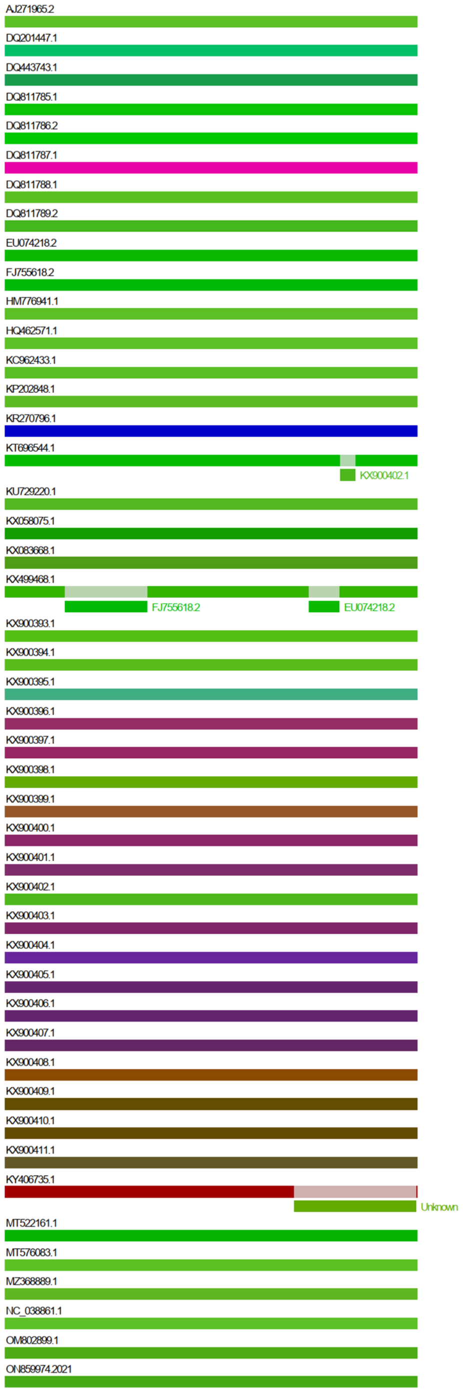

Figure S4 The recombination analysis of PDCoV full genomes by RDP4.

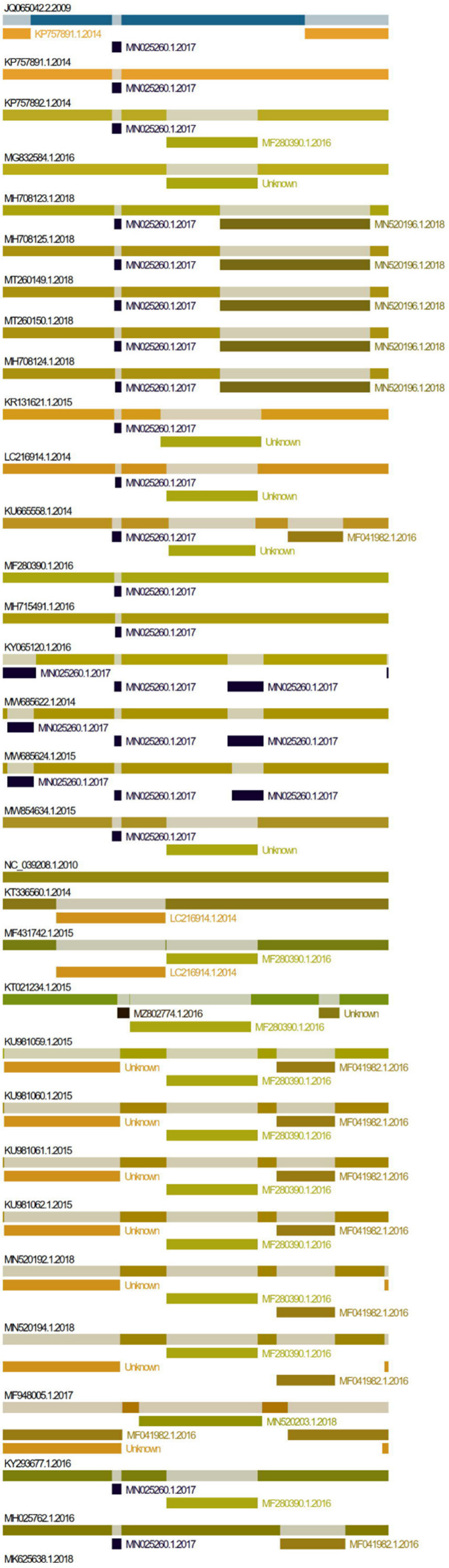
JQ065042.2.2009
KP757891.1.2014
MN025260.1.2017
KP757891.1.2014
MN025260.1.2017
KP757892.1.2014
MN025260.1.2017
MF280390.1.2016
MG832584.1.2016
Unknown
MH708123.1.2018
MN025260.1.2017
MN520196.1.2018
MH708125.1.2018
MN025260.1.2017
MN520196.1.2018
MT260149.1.2018
MN025260.1.2017
MN520196.1.2018
MT260150.1.2018
MN025260.1.2017
MN520196.1.2018
MH708124.1.2018
MN025260.1.2017
MN520196.1.2018
KR131621.1.2015
MN025260.1.2017
Unknown
LC216914.1.2014
MN025260.1.2017
Unknown
KU665558.1.2014
MN025260.1.2017
MF041982.1.2016
Unknown
MF280390.1.2016
MN025260.1.2017
MH715491.1.2016
MN025260.1.2017
KY065120.1.2016
MN025260.1.2017
MN025260.1.2017
MN025260.1.2017
MW685622.1.2014
MN025260.1.2017
MN025260.1.2017
MN025260.1.2017
MW685624.1.2015
MN025260.1.2017
MN025260.1.2017
MN025260.1.2017
MW854634.1.2015
MN025260.1.2017
Unknown
NC_039208.1.2010
KT336560.1.2014
LC216914.1.2014
MF431742.1.2015
MF280390.1.2016
LC216914.1.2014
KT021234.1.2015
MZ802774.1.2016
Unknown
MF280390.1.2016
KU981059.1.2015
Unknown
MF041982.1.2016
MF280390.1.2016
KU981060.1.2015
Unknown
MF041982.1.2016
MF280390.1.2016
KU981061.1.2015
Unknown
MF041982.1.2016
MF280390.1.2016
KU981062.1.2015
Unknown
MF041982.1.2016
MF280390.1.2016
MN520192.1.2018
Unknown
MF280390.1.2016
MF041982.1.2016
MN520194.1.2018
MF280390.1.2016
Unknown
MF041982.1.2016
MF948005.1.2017
MN520203.1.2018
MF041982.1.2016
Unknown
KY293677.1.2016
MN025260.1.2017
MF280390.1.2016
MH025762.1.2016
MN025260.1.2017
MF041982.1.2016
MK625638.1.2018

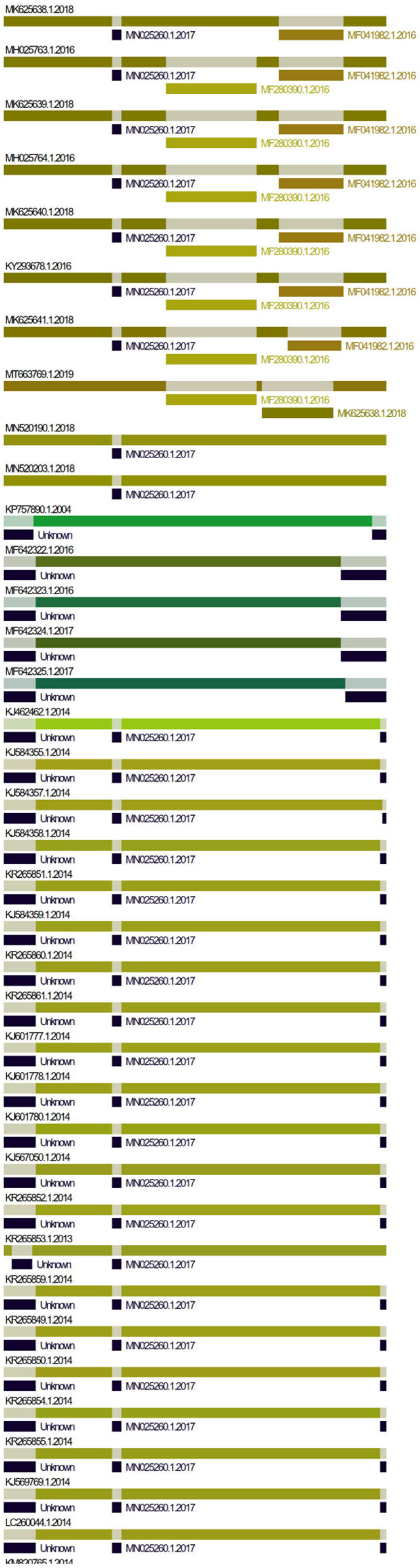

MK625638.1.2018
MN025260.1.2017
MF041982.1.2016
MH025763.1.2016
MN025260.1.2017
MF041982.1.2016
MF280390.1.2016
MK625639.1.2018
MN025260.1.2017
MF041982.1.2016
MF280390.1.2016
MH025764.1.2016
MN025260.1.2017
MF041982.1.2016
MF280390.1.2016
MK625640.1.2018
MN025260.1.2017
MF041982.1.2016
MF280390.1.2016
KY293678.1.2016
MN025260.1.2017
MF041982.1.2016
MF280390.1.2016
MK625641.1.2018
MN025260.1.2017
MF041982.1.2016
MF280390.1.2016
MT663769.1.2019
MF280390.1.2016
MK625638.1.2018
MN520190.1.2018
MN025260.1.2017
MN520203.1.2018
MN025260.1.2017
KP757890.1.2004
Unknown
MF642322.1.2016
Unknown
MF642323.1.2016
Unknown
MF642324.1.2017
Unknown
MF642325.1.2017
Unknown
KJ462462.1.2014
Unknown
MN025260.1.2017
KJ584355.1.2014
Unknown
MN025260.1.2017
KJ584357.1.2014
Unknown
MN025260.1.2017
KJ584358.1.2014
Unknown
MN025260.1.2017
KR265851.1.2014
Unknown
MN025260.1.2017
KJ584359.1.2014
Unknown
MN025260.1.2017
KR265860.1.2014
Unknown
MN025260.1.2017
KR265861.1.2014
Unknown
MN025260.1.2017
KJ601777.1.2014
Unknown
MN025260.1.2017
KJ601778.1.2014
Unknown
MN025260.1.2017
KJ601780.1.2014
Unknown
MN025260.1.2017
KJ567050.1.2014
Unknown
MN025260.1.2017
KR265852.1.2014
Unknown
MN025260.1.2017
KR265853.1.2013
Unknown
MN025260.1.2017
KR265859.1.2014
Unknown
MN025260.1.2017
KR265849.1.2014
Unknown
MN025260.1.2017
KR265850.1.2014
Unknown
MN025260.1.2017
KR265854.1.2014
Unknown
MN025260.1.2017
KR265855.1.2014
Unknown
MN025260.1.2017
KJ569769.1.2014
Unknown
MN025260.1.2017
LC260044.1.2014
Unknown
MN025260.1.2017

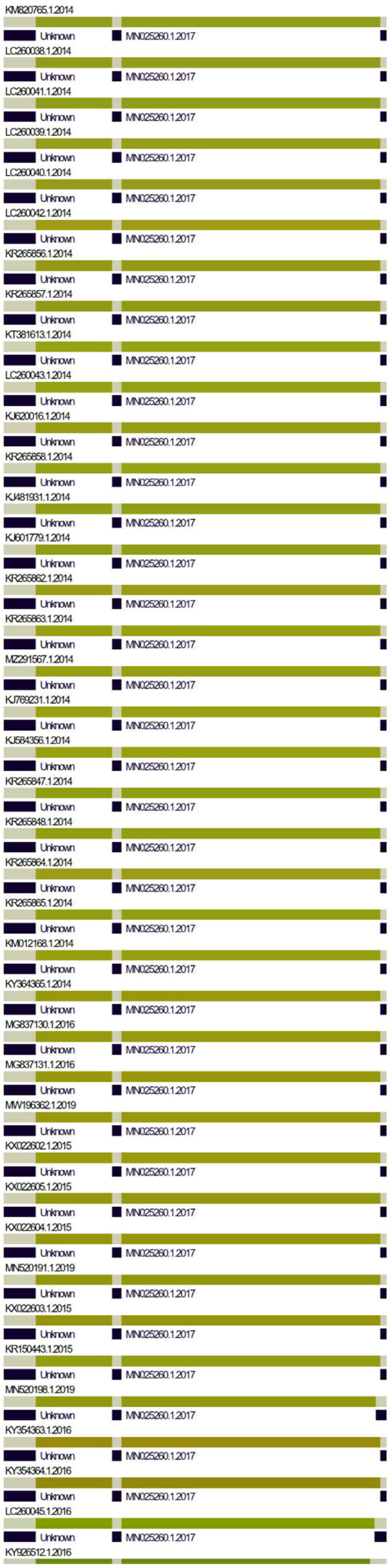
KM820765.1.2014
Unknown
MN025260.1.2017
LC260038.1.2014
Unknown
MN025260.1.2017
LC260041.1.2014
Unknown
MN025260.1.2017
LC260039.1.2014
Unknown
MN025260.1.2017
LC260040.1.2014
Unknown
MN025260.1.2017
LC260042.1.2014
Unknown
MN025260.1.2017
KR265856.1.2014
Unknown
MN025260.1.2017
KR265857.1.2014
Unknown
MN025260.1.2017
KT381613.1.2014
Unknown
MN025260.1.2017
LC260043.1.2014
Unknown
MN025260.1.2017
KJ620016.1.2014
Unknown
MN025260.1.2017
KR265858.1.2014
Unknown
MN025260.1.2017
KJ481931.1.2014
Unknown
MN025260.1.2017
KJ601779.1.2014
Unknown
MN025260.1.2017
KR265862.1.2014
Unknown
MN025260.1.2017
KR265863.1.2014
Unknown
MN025260.1.2017
MZ291567.1.2014
Unknown
MN025260.1.2017
KJ769231.1.2014
Unknown
MN025260.1.2017
KJ584356.1.2014
Unknown
MN025260.1.2017
KR265847.1.2014
Unknown
MN025260.1.2017
KR265848.1.2014
Unknown
MN025260.1.2017
KR265864.1.2014
Unknown
MN025260.1.2017
KR265865.1.2014
Unknown
MN025260.1.2017
KM012168.1.2014
Unknown
MN025260.1.2017
KY364365.1.2014
Unknown
MN025260.1.2017
MG837130.1.2016
Unknown
MN025260.1.2017
MG837131.1.2016
Unknown
MN025260.1.2017
MW196362.1.2019
Unknown
MN025260.1.2017
KX022602.1.2015
Unknown
MN025260.1.2017
KX022605.1.2015
Unknown
MN025260.1.2017
KX022604.1.2015
Unknown
MN025260.1.2017
MN520191.1.2019
Unknown
MN025260.1.2017
KX022603.1.2015
Unknown
MN025260.1.2017
KR150443.1.2015
Unknown
MN025260.1.2017
MN520198.1.2019
Unknown
MN025260.1.2017
KY354363.1.2016
Unknown
MN025260.1.2017
KY354364.1.2016
Unknown
MN025260.1.2017
LC260045.1.2016
Unknown
MN025260.1.2017
KY926512.1.2016

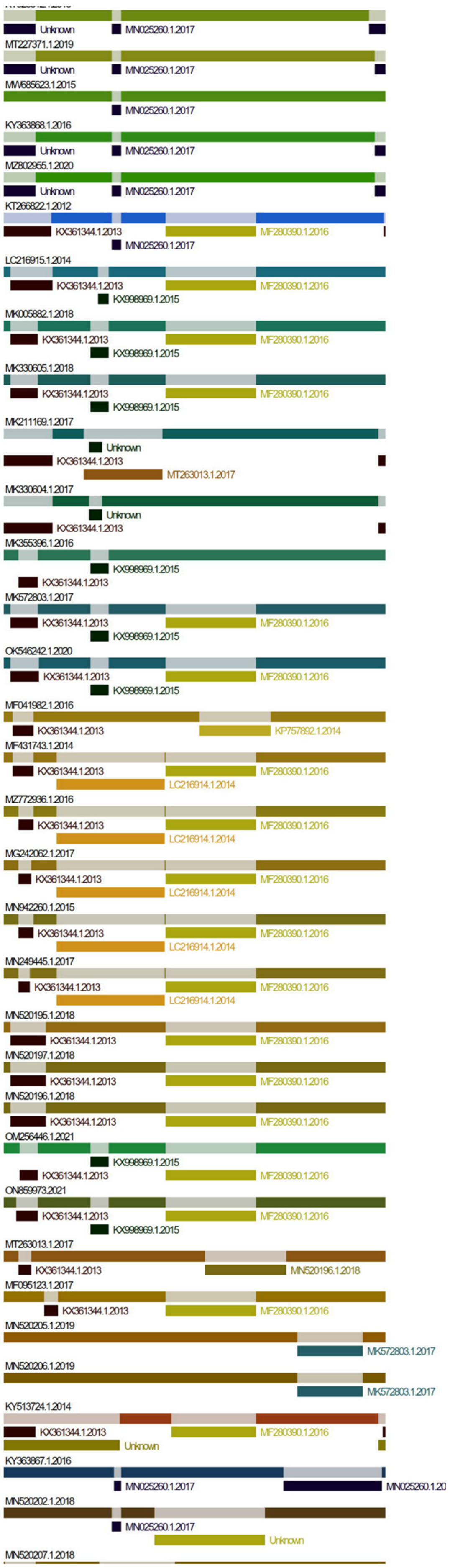

Unknown
MN025260.1.2017
MT227371.1.2019
Unknown
MN025260.1.2017
MW685623.1.2015
MN025260.1.2017
KY363868.1.2016
Unknown
MN025260.1.2017
MZ802955.1.2020
Unknown
MN025260.1.2017
KT266822.1.2012
KX361344.1.2013
MF280390.1.2016
MN025260.1.2017
LC216915.1.2014
KX361344.1.2013
MF280390.1.2016
KX998969.1.2015
MK005882.1.2018
KX361344.1.2013
MF280390.1.2016
KX998969.1.2015
MK330605.1.2018
KX361344.1.2013
MF280390.1.2016
KX998969.1.2015
MK211169.1.2017
Unknown
KX361344.1.2013
MT263013.1.2017
MK330604.1.2017
Unknown
KX361344.1.2013
MK355396.1.2016
KX998969.1.2015
KX361344.1.2013
MK572803.1.2017
KX361344.1.2013
MF280390.1.2016
KX998969.1.2015
OK546242.1.2020
KX361344.1.2013
MF280390.1.2016
KX998969.1.2015
MF041982.1.2016
KX361344.1.2013
KP757892.1.2014
MF431743.1.2014
KX361344.1.2013
MF280390.1.2016
LC216914.1.2014
MZ772936.1.2016
KX361344.1.2013
MF280390.1.2016
LC216914.1.2014
MG242062.1.2017
KX361344.1.2013
MF280390.1.2016
LC216914.1.2014
MN942260.1.2015
KX361344.1.2013
MF280390.1.2016
LC216914.1.2014
MN249445.1.2017
KX361344.1.2013
MF280390.1.2016
LC216914.1.2014
MN520195.1.2018
KX361344.1.2013
MF280390.1.2016
MN520197.1.2018
KX361344.1.2013
MF280390.1.2016
MN520196.1.2018
KX361344.1.2013
MF280390.1.2016
OM256446.1.2021
KX998969.1.2015
KX361344.1.2013
MF280390.1.2016
ON859973.2021
KX361344.1.2013
MF280390.1.2016
KX998969.1.2015
MT263013.1.2017
KX361344.1.2013
MN520196.1.2018
MF095123.1.2017
KX361344.1.2013
MF280390.1.2016
MN520205.1.2019
MK572803.1.2017
MN520206.1.2019
MK572803.1.2017
KY513724.1.2014
KX361344.1.2013
MF280390.1.2016
Unknown
KY363867.1.2016
MN025260.1.2017
MN025260.1.20
MN520202.1.2018
MN025260.1.2017
Unknown
MN520207.1.2018

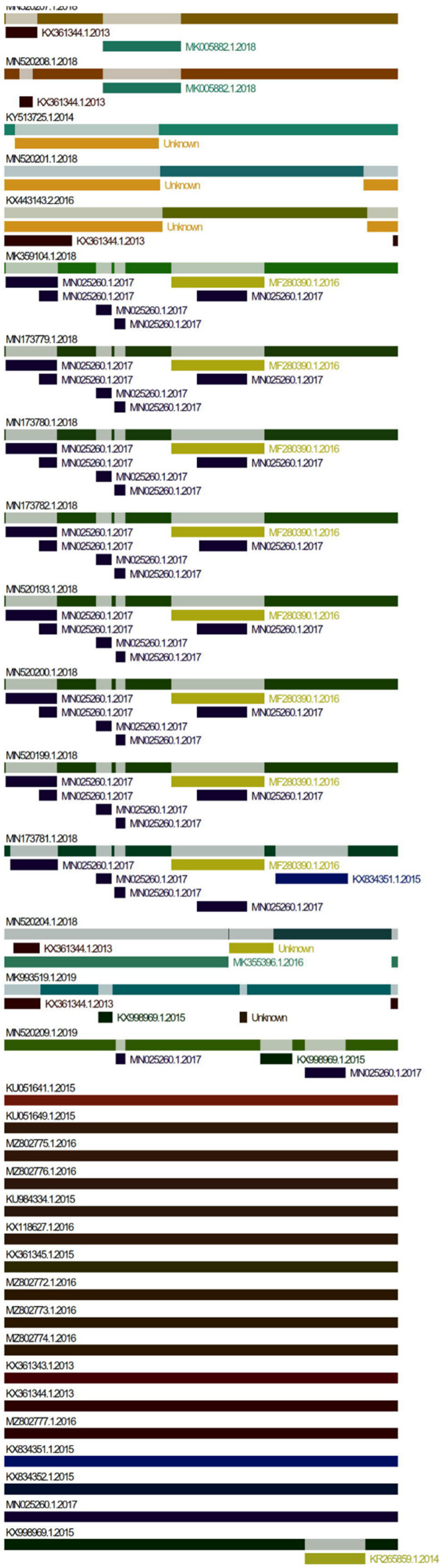
KX361344.1.2013
MK005882.1.2018
MN520208.1.2018
MK005882.1.2018
KX361344.1.2013
KY513725.1.2014
Unknown
MN520201.1.2018
Unknown
KX443143.2.2016
Unknown
KX361344.1.2013
MK359104.1.2018
MN025260.1.2017
MF280390.1.2016
MN025260.1.2017
MN025260.1.2017
MN025260.1.2017
MN025260.1.2017
MN173779.1.2018
MN025260.1.2017
MF280390.1.2016
MN025260.1.2017
MN025260.1.2017
MN025260.1.2017
MN025260.1.2017
MN173780.1.2018
MN025260.1.2017
MF280390.1.2016
MN025260.1.2017
MN025260.1.2017
MN025260.1.2017
MN025260.1.2017
MN173782.1.2018
MN025260.1.2017
MF280390.1.2016
MN025260.1.2017
MN025260.1.2017
MN025260.1.2017
MN025260.1.2017
MN520193.1.2018
MN025260.1.2017
MF280390.1.2016
MN025260.1.2017
MN025260.1.2017
MN025260.1.2017
MN025260.1.2017
MN520200.1.2018
MN025260.1.2017
MF280390.1.2016
MN025260.1.2017
MN025260.1.2017
MN025260.1.2017
MN025260.1.2017
MN520199.1.2018
MN025260.1.2017
MF280390.1.2016
MN025260.1.2017
MN025260.1.2017
MN025260.1.2017
MN025260.1.2017
MN173781.1.2018
MN025260.1.2017
MF280390.1.2016
MN025260.1.2017
KX834351.1.2015
MN025260.1.2017
MN025260.1.2017
MN520204.1.2018
KX361344.1.2013
Unknown
MK355396.1.2016
MK993519.1.2019
KX361344.1.2013
KX998969.1.2015
Unknown
MN520209.1.2019
MN025260.1.2017
KX998969.1.2015
MN025260.1.2017
KU051641.1.2015
KU051649.1.2015
MZ802775.1.2016
MZ802776.1.2016
KU984334.1.2015
KX118627.1.2016
KX361345.1.2015
MZ802772.1.2016
MZ802773.1.2016
MZ802774.1.2016
KX361343.1.2013
KX361344.1.2013
MZ802777.1.2016
KX834351.1.2015
KX834352.1.2015
MN025260.1.2017
KX998969.1.2015
KR265859.1.2014
